# Health-Related Social Control over Physical Activity: Interactions with Age and Sex

**DOI:** 10.1155/2012/321098

**Published:** 2012-06-19

**Authors:** Kelly A. Cotter

**Affiliations:** Department of Psychology, Sacramento State University, 6000 J Street, Sacramento, CA 95819, USA

## Abstract

Despite the disease prevention benefits of engaging in life-long regular physical activity, many adults remain sedentary. The social environment provides an important context for health and health behavior across the lifespan, as well as a potential point of intervention for increasing physical activity. Self-reports of perceived social support, social strain, positive social control, and negative social control were examined for their cross-sectional relationships to physical activity frequency in purposive samples of younger and older adults (*N* = 371, ages from 18 to 97, 68% women). Hierarchical regression analyses revealed that perceived support and perceived strain were not correlated with physical activity. However, age and sex interacted with social control, such that more positive social control was associated with more frequent physical activity for younger men. Furthermore, more positive and negative social control were significantly associated with less frequent physical activity for older men, while social control was not associated with physical activity among women. While younger men may be encouraged toward healthier behaviors by positive social control messages, social control attempts may backfire when targeting older men. Implications for physical activity promotion are discussed.

## 1. Introduction

Engaging in regular physical activity is a powerful tool for maintaining health and reducing disability across the lifespan [1]. The Centers for Disease Control and Prevention (CDC, [2]) recommend that all adults, including older adults, should accumulate *at least* 150 minutes of moderate-intensity aerobic activity (e.g., brisk walking) or 75 minutes of vigorous-intensity aerobic activity (e.g., running) on most days of the week. An equivalent combination of moderate and vigorous aerobic activity is also acceptable. In addition to strengthening the cardiovascular system through aerobic activity, the CDC also recommends that adults participate in strength-building activity on at least two days per week. Importantly, these activity bouts can be broken into 10-minute sessions and spread throughout the week. Furthermore, these activity bouts do not have to be planned, structured exercise sessions; Leisure activities like gardening or bowling also “count” toward the accumulation of health-enhancing activity minutes, as long as the individual participating in the activity perceives it to be moderately or vigorously intense.

The CDC's physical activity recommendations are based on research demonstrating that physical activity effectively reduces risk for age-related diseases including diabetes, heart disease, cerebrovascular disease, osteoarthritis, and some cancers [3]. In addition, greater physical activity is associated with more strength and balance, relief of many disease symptoms, and increased longevity [3]. Due to its profound health effects, it is imperative to understand how to promote physical activity behavior across the lifespan. The social landscape provides one important context for physical activity behavior [1], and it also changes in meaningful ways for men and women as they age [4]. Thus, in the present study, I examined the direct and moderated relationships of physical activity to perceived social support, perceived social strain, positive social control, and negative social control.

Perceived social support, defined by Walen and Lachman as “one's perceived notion of the caring and understanding exhibited by the [social] network” [5, page 7], has a consistent positive influence on health and health behaviors [6]. On the other hand, perceived strain, defined as “individuals' general perception of the critical, irritating, and unreliable nature of their network” [5, page 7], typically has negative implications for health and health behaviors [7].

The structure of the social network, and thus the availability of support and strain, changes in meaningful ways as people age. The social network narrows in later life, such that older adults maintain or increase contact with intimates (including spouses and close friends) but reduce contact with acquaintances [8]. This pattern is consistent with socioemotional selectivity theory [4], which states that people are motivated by emotional goals when they perceive time as limited. Applying the sense of time-sensitivity to the lifespan, older adults (relative to younger adults) are more motivated to maintain bonds with sources of support and sever ties with sources of strain because older adults are closer to the end of the lifespan and thus more motivated to induce positive emotional states [8]. Therefore, older adults should report more support and less strain from their social partners than do younger adults. Consistent with this assertion, Walen and Lachman [5] found that older adults (age 60–75) reported more family support and less family strain than younger adults (age 25–39) in a sample of married or cohabitating adults. Furthermore, older men reported more support from their spouse than younger men [5].

In addition to predicting health directly, perceived support is also linked to greater physical activity participation [1]. Furthermore, although perceived strain is associated with poor health outcomes, it has a positive relationship with physical activity. For example, in a large national survey of adults aged 35 to 84 years, participants who reported more perceived strain also reported more frequent physical activity [1]. The relationship between perceived strain and physical activity was moderated by age, such that it was stronger for older adults. To explain the counterintuitive relationship between strain and physical activity, some authors have suggested that people may use social sanctions to elicit desired behavior in social partners [9]. These interactions, while potentially well intended and effective, may be perceived as straining and controlling. Indeed, health-related social control, broadly defined as the efforts of network members to change a target's health behavior [10], provides a potential pathway to health.

At the most basic level, health-related social control is theorized to act as the mechanism by which network members influence health, such that someone in the social network exerts social control, which causes the recipient to change his or her targeted health behavior, which in turn affects the recipient's health [11]. These control attempts can be perceived as positive (e.g., encouragement) or negative (e.g., criticism) [12]. For example, a person who wants to support a friend by including her in activities might invite her to a water aerobics class. This friend may interpret such support as encouragement for healthy behaviors (positive social control), or she might feel nagged to exercise (negative social control). Thus, social control may explain the relationship of perceived support to health behaviors, such that social control provides one of many strategies to offer support [1]. In fact, social control and general perceived support are moderately correlated [13].

It is important to point out that general perceived support is not the same concept as domain-specific social support [13]: general support refers to a person's global perceptions about his or her available supports, while domain-specific support refers to a person's perceptions of available support or actual support received given specific circumstances. Domain-specific support has received a lot of attention in the physical activity literature. Studies show that greater exercise-support or physical activity-support predict greater exercise or physical activity participation [14]. Domain-specific support is the same concept as positive social control (i.e., encouragement for physical activity participation), but the terms used to describe the concept have differed in the literature. I use the social control terminology in this paper.

An overwhelming majority of research has found that positive social control is linked to health behavior change [15], including physical activity [16], while negative social control is associated with resistance to behavior change [17]. However, the influence of social control may change across the life course. For example, Tucker et al. found that older adults reported fewer people in their networks who provided social control than younger adults [18]. Additionally, older adults who reported more positive affect from social control attempts were more likely to hide unhealthy behaviors from the social network [19]. Tucker et al. argue that older adults want to maintain harmony with the social network but may be unwilling to give up negative health behaviors, so instead they hide these behaviors [19]. This explanation is consistent with socioemotional selectivity theory, which states that older adults are more motivated than younger adults to satisfy emotional goals, like maintaining harmony with the social network [4]. Tucker and colleagues' results suggest that positive social control may not necessarily promote health behavior among older adults, particularly if they enjoy high-quality relationships [19].

 In addition to being less effective for older adults than for younger adults, social control attempts may also be less effective for women than for men. Men are more likely than women to be the targets of social control in marriages [20], and wives are more successful social control agents than husbands [21]. Even outside of the marital relationship, social control attempts from the partner, family, and friends were effective at reducing men's smoking behavior over four months but had no effect on women's smoking [22]. Taken together, previous research suggests that social control may not be an effective strategy for changing the health behaviors of older adults or women. However, social control may be successful when targeting the health behaviors of younger men. Because younger men might react differently to social control than older men or women react, the potential interaction between age, sex, and social control was explored in the present study.

The present study was designed to examine the links between physical activity behavior, perceived support and strain, and health-related social control. As argued above, positive social control shares conceptual similarities to social support and may even act as a method of providing emotional, informational, and instrumental support [13]. While research has demonstrated the independence of social control from social support [13], social control has not yet been examined for its independence from social strain, despite conceptual similarities. In fact, Cotter and Lachman have argued that social partners may intend to be supportive with their health-related messages, but that recipients of those messages may perceive such interactions as straining [1]. Therefore, from the perspective of the target, strain may share an even stronger relationship to social control than support does. Examining all four forms of social influence together is imperative for informing health behavior interventions because this will identify which type has the strongest relationship to health behavior, and thus the most appropriate target for intervention.

Based on the results of previous studies, I predicted that more frequent physical activity would be associated with more perceived support and strain, and that the positive relationship of strain to physical activity would be stronger among older adults than among younger adults [1]. I also explored a potential interaction between age and sex in the context of health-related social control. Based on previous research revealing that social control was ineffective when targeting older adults [19] and women [22], I predicted that age and sex would moderate the relationship of social control to physical activity in the present study, such that more positive and less negative social control would be associated with more frequent physical activity for younger men, but that social control would not be related to physical activity for older adults and for women.

## 2. Method

### 2.1. Procedure

Data for the present study were simultaneously collected from two different sources: undergraduate psychology majors at a northern California university and community-dwelling older adults (who were recruited through flyers and presentations at local senior centers). Upon volunteering, participants were guided through an informed consent procedure. Next, participants completed the survey. A research assistant was available to answer questions as the participants completed the survey. Finally, participants were debriefed and given information about psychological and fitness resources. Undergraduates received course research credit for their participation, and older adults were entered into a raffle for one of fifteen $25 gift cards. The protocol received institutional approval for the ethical treatment of participants.

### 2.2. Participants

Participants were 371 noninstitutionalized, English-speaking adults ages from 18 to 97 (*M* = 45.28, SD = 26.93), who volunteered to participate in a paper-and-pencil survey. The sample recruited through the university ranged between 18 and 52 years old (*M* = 22.02, SD = 4.46), while the sample recruited through senior centers ranged between 46 and 97 years old (*M* = 73.71, SD = 10.72). Information regarding the demographic characteristics of race/ethnicity, marital status, education, and income was also collected (see Table 1), and each of these variables was dichotomized for analyses.

For the race/ethnicity variable, White participants comprised one group (51.3%) and all other races were combined to create the non-White group (48.7%). Marital status was recoded with one category of participants who were currently married (17%) and the second category with participants who were separated, divorced, widowed, or never married (83%). Education was dichotomized with one group of participants who had earned up to a high school diploma or GED (22.9%) and the other group who attended some college or higher education (77.1%). Finally, annual household income was recoded such that participants earning $20,000 or less comprised one group (50%) and participants earning $20,001 or more comprised the other group (50%).

The undergraduate sample reflects an accurate representation of the age and ethnicity of the undergraduate population of the university. However, women are overrepresented [23]. Older women, African Americans, and people of multiethnic heritage are overrepresented in the older adult sample compared to the population of American older adults [24, 25]. Furthermore, it should be presumed that the older adults sampled for the present study represent a relatively healthy and active segment of the older adult population. While participants were not asked specific questions regarding health conditions or pain; all were healthy enough to either attend classes at a university or to attend functions and activities at a community center.

### 2.3. Measures 

#### 2.3.1. Perceived Support and Strain

Responses to questions reflecting perceived social support (e.g., “How much do your friends really care about you?”) and perceived social strain (e.g., “How often does your family criticize you?”) from the spouse/partner, family, and friends were averaged across source [5], with higher scores reflecting more support (*M* = 3.45, SD = .48, *α* = .84) or strain (*M* = 1.92, SD = .58, *α* = .85). Although only 17% of the sample reported being married, most participants (54%) had a significant other to which they referred on items regarding partner support and strain. The range of scores in the present sample reflects the possible range (1 = Not at all to 4 = A lot) and published reliability (*α* range from  .79 to  .91) [5]. 

#### 2.3.2. Social Control over Physical Activity

 Social control over physical activity was measured as positive and negative social control from the spouse/partner, family, and friends. The positive social control scale (e.g., “How much does your partner encourage you to exercise?”) was adapted from Sallis et al. [26], who reported high test-retest and internal consistency reliability for the measure. Responses to items were averaged, with higher scores reflecting more positive social control (*M* = 2.39, SD = .99, *α* = .97). The range of scores in the present sample reflects the possible range (1 = Never to 5 = Very Often). 

The negative social control measure was created for the present study to measure negative social control from the spouse/partner, family, and friends aimed at promoting exercise behavior. Modeled after the positive social control measure [26], respondents were asked to report how often their spouse/partner, family members not including the spouse or partner, or friends did each of the following in the past month: nag you about exercise, demand that you exercise on recreational outings, demand that you discuss exercise, tell you ideas on how you can get more exercise, make negative comments about your physical appearance, pressure you to exercise, and make remarks about how much you should be exercising. Participants responded on a five-point scale (1 = Never, 2 = Rarely, 3 = Sometimes, 4 = Often, 5 = Very Often). 

A principal components factor analysis was conducted on each subscale, and each subscale had an eigenvalue above 2. The factor solution for the partner domain included one factor that accounted for 53.74% of the variance with an eigenvalue of 3.76 (*α* = .84). The factor solution for the family domain included one factor that accounted for 55.56% of the variance with an eigenvalue of 3.89 (*α* = .86). The factor solution for the friend domain included one factor that accounted for 45.97% of the variance with an eigenvalue of 3.22 (*α* = .77). Responses were averaged across social partner for the present study, with possible scores ranging from 1 to 5. Higher scores reflect more negative social control (range = 1–5, *M* = 1.53, SD = .56, *α* = .89). 

#### 2.3.3. Physical Activity

Physical activity was measured with 9 items assessing participants' frequency of vigorous and moderate physical activity on a six-point scale ranging from 0 (Never) to 5 (Several times a week). Participants' scores reflected the possible range in the present sample. Based on Cotter and Lachman's measure [1], the setting in which the participant was most active (work, home, or leisure) comprised the participants' vigorous and moderate scores. In other words, the highest “moderate” score of the three settings was used as the indicator of frequency of moderate physical activity, and the highest “vigorous” score of the three settings was used as the indicator of frequency of vigorous physical activity. In this manner, if the participant performed regular activity in the home but not at work or for leisure, the respondent was still classified as regularly active. Next, the higher of the moderate versus vigorous scores was used as the indicator of total physical activity frequency (*M* = 3.89, SD = 1.59). Moderate (*M* = 3.77, SD = 1.63) and vigorous (*M* = 3.10, SD = 1.88) activity were highly correlated with each other in the present study: *r* = .66, *P* < .001. Cotter and Lachman [1] reported that calculating the physical activity score in this manner yields the best approximation possible to data from the Centers for Disease Control and Prevention [2], which recommends that adults accumulate at least 30 minutes of moderate aerobic activity on most days of the week or 20 minutes of vigorous aerobic activity three days per week. 

## 3. Results

First, analyses were conducted on all variables to ensure normality of the distribution and reliability of measures. The negative social control measure was positively skewed and was transformed using a log 10 transformation. Next, exploratory analyses were conducted to determine whether there were different patterns of results based on different sources of influence (partner, family, and friends). Patterns were consistent between the partner, family, and friend domains. Thus, social influence variable scores were averaged across the domains, as described above in the measures section. All continuous independent variables were centered, and 2- and 3-way interaction terms were calculated by multiplying the centered support, strain, positive social control, or negative social control score by sex (0 = male, 1 = female) and age (0 = younger, 1 = older). Age was dichotomized at the mean of 45 years because the sampling technique led to a very bimodal distribution (*n* = 205 for younger adults, *n* = 166 for older adults, see Table 1). 

Zero-order correlations between all variables were calculated and examined (see Table 2). Next, the direct and moderated cross-sectional relationships with physical activity frequency were examined in Hierarchical Multiple Regression (HMR) analyses using pairwise deletion and the MODPROBE procedure for SPSS developed by Hayes and Matthes [27]. Interactions with positive social control and negative social control were each examined in separate analyses in order to reduce problems associated with multicollinearity. Thus, age, sex, perceived support, perceived strain, positive social control, and negative social control were entered on Step 1 of both HMR analyses. Two-way age, sex, and *positive* social control interaction terms were entered on Step 2 of Model 1 (presented in the top half of Table 3), and two-way age, sex, and *negative* social control interaction terms were entered on Step 2 of Model 2 (presented in the bottom half of Table 3). The three-way interaction term age-by-sex-positive social control was entered on Step 3 of Model 1, and the three-way interaction term age-by-sex-by-negative social control was entered on Step 3 of Model 2. The Johnson-Neyman technique was used in follow-up analyses to examine regions of significance for the interactions [27]. 

Regression analyses revealed that none of the demographic characteristics had a significant relationship to physical activity when age and sex were in the models, so demographic characteristics were trimmed from the analyses to conserve statistical power. Furthermore, consistent with the bivariate relationships presented in Table 2, regression analyses revealed no statistically significant direct or moderated relationships between perceived social support and perceived social strain with physical activity. Thus, the three-way interaction terms of age-by-sex-by-perceived support and age-by-sex-by-perceived strain were also trimmed from the reported models to conserve power, but are available upon request. The results of the regression analyses are shown in Table 3, with interactions involving positive social control presented in the top half of the table and interactions involving negative social control presented in the bottom half of the table. 

### 3.1. Positive Social Control

**Figure 1:**
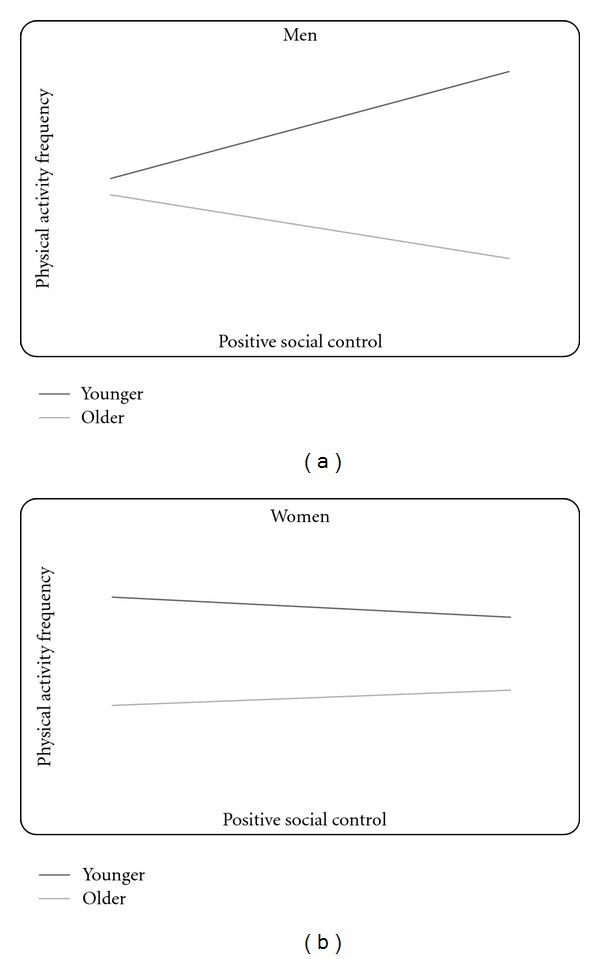
(a) The interaction of age and positive social control on physical activity for men. (b) The interaction of age and positive social control on physical activity for women. *Note:* the figures show predicted regression lines for one standard deviation above (high) and one standard deviation below (low) the mean of positive social control. The simple slope is statistically significant for older men.

Variables in the model examining positive social control explained 20.1% of the total variance in physical activity frequency,* F* (10, 262) = 6.60, *P* < .001, and revealed a significant 3-way interaction between age, sex, and positive social control (*β* = .27, *P* = .05, see top half of Table 3) even after controlling for the relationships of perceived support, perceived strain, and negative social control. To plot the interaction, the data were split by sex and the 2-way interactions of age and positive social control were examined separately for men and women. The figures show predicted regression lines for younger and older adults at one standard deviation below (low) and one standard deviation above (high) the mean of positive social control. As shown in Figure 1, the 2-way age-by-positive social control interaction was significant among men, Δ*R* ^2^ = .10, *F* (1, 73) = 9.85, *P* < .001. Follow-up analyses used the Johnson-Neyman technique to determine regions of significance within the interactive effect [27]. Specifically, the effect of positive social control at each level of age (younger versus older) was examined among men and women. The follow-up analyses revealed that more positive social control was associated with more frequent physical activity for younger men (*t* = 1.67, *P* = .10), while more positive social control was associated with significantly less frequent physical activity for older men (*t* = −2.30, *P* = .03). The interaction was not significant for women, Δ*R* ^2^ = .001, *F* (1, 174) =.31, *P* = .58. Instead, only the direct relationship of age and physical activity was statistically significant, such that younger age was related to more frequent physical activity (**β** = −.37, *P* < .001). 

### 3.2. Negative Social Control

**Figure 2:**
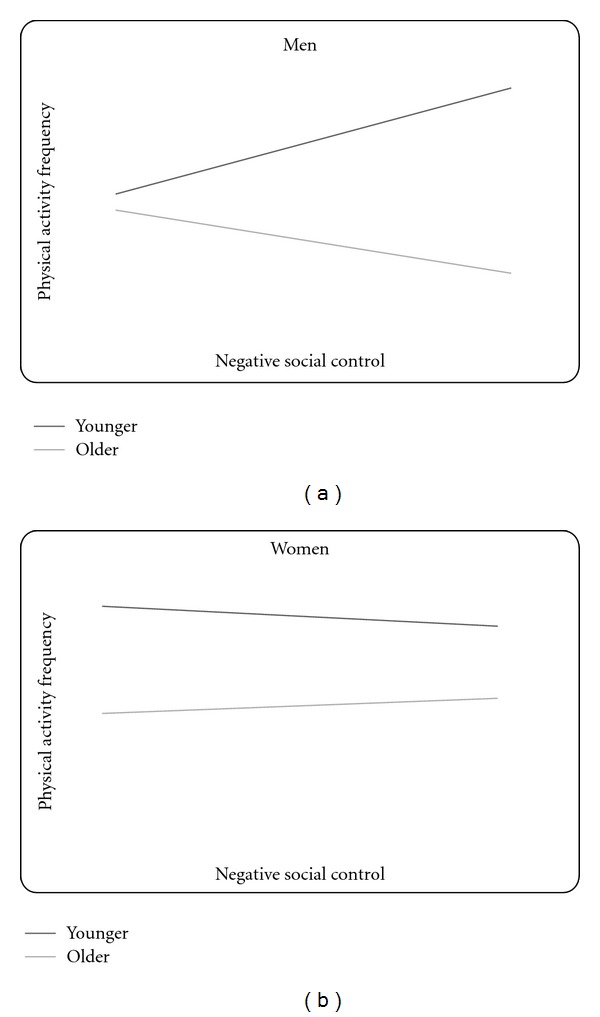
(a) The interaction of age and negative social control on physical activity for men. (b) The interaction of age and negative social control on physical activity for women. *Note:* the figures show predicted regression lines for one standard deviation above (high) and one standard deviation below (low) the mean of negative social control. The simple slope is statistically significant for older men.

Variables in the model examining negative social control explained 18.3% of the total variance in physical activity frequency,* F* (10, 261) = 7.09, *P* < .001, and revealed a significant 3-way interaction between age, sex, and negative social control (*β* = .41, *P* = .001, see bottom half of Table 3) even after controlling for the relationships of perceived support, perceived strain, and positive social control. The interaction was plotted following the same method described above (see Figure 2), and further analysis using the Johnson Neyman technique [27] revealed that the 2-way age-by-negative social control interaction was significant among men, Δ*R* ^2^ = .09, *F* (1, 73) = 8.57, *P* = .005, such that more negative social control was associated with less frequent physical activity for older men (*t* = −2.52, *P* = .01), while negative social control was not significantly associated with physical activity for younger men (*t* = .90, *P* = .37). Again here, the interaction was not significant for women, Δ*R* ^2^ = .003, *F*(1,174) = .66, *P* = .42. Instead, only the direct relationship of younger age and more frequent physical activity was statistically significant for women (**β** = −.38, *P* < .001). 

## 4. Discussion

 Younger age was associated with more frequent physical activity in all analyses. This is consistent with a large body of research demonstrating younger adults as more active than older adults [28]. Younger adults are less likely than older adults to suffer from disabling conditions [29] and are more aware of the benefits of exercise [30]. Thus, younger adults may have more opportunity to be active. 

While the main effect of age on physical activity is important for public health intervention, the present study is among the first to examine the interactive effect of age, sex, and social control on physical activity. As predicted, age and sex moderated the relationship of social control to physical activity, such that more positive social control was associated with more frequent physical activity for younger men (albeit at trend-level statistical significance). These results are consistent with results from a sample of men living with HIV, demonstrating that positive social control from friends, family members, and romantic partners was associated with behavior change [17]. Because the current data are cross-sectional, we cannot determine if young men engage in health behaviors in response to social control attempts or, alternatively, if they receive social control in response to health behaviors. However, based on previous experimental research where social control predicted health behavior change [31], the social control to behavior direction seems more likely than the opposite. 

In contrast to younger men, older men in the present study reported significantly less physical activity when they perceived more social control. These results do not support my hypothesis that social control would not affect older men. This hypothesis was based on a study from Tucker and colleagues, who found that older adults who reported more positive affect from social control attempts were more likely to hide unhealthy behaviors from the social network [19]. Consistent with socioemotional selectivity theory [4], Tucker et al. argued that older adults want to maintain harmony with the social network, but may be unwilling to give up negative health behaviors, so instead they hide these behaviors from their loved ones. 

Alternatively, older men may suffer from more health conditions that benefit from engaging in physical activity. For example, older men are more likely than younger men to suffer from heart disease [32], a chronic illness that can be controlled through regular physical activity [33]. Social partners may engage in health-related social control in an attempt to encourage illness-management [13], but heart disease also makes physical activity more difficult to complete [33]. Thus older men may actively refrain from engaging in this health behavior, despite the social control attempts of their network members, because they are unable or unwilling to begin or maintain a physical activity regimen. In addition to health, response bias provides another potential explanation for the negative relationship between physical activity and social control: older men may perceive more social control from their social partner, or they may be more willing to report social control than younger men [20]. 

Consistent with previous research [22] and with hypotheses, social control had no relationship to physical activity among women in the present study. Westmaas et al. suggest that receiving social control may undermine women's perceptions of themselves as the health-keepers of their families and as positive role models for health behaviors [22]. Westmaas et al. explain that this psychological burden may interfere with women's ability to change their health behavior. Thus, women may need to bolster their cognitive and emotional resources (i.e., self-efficacy) before attempting a physical activity behavior change. Because older adults have low confidence in their ability to adopt and maintain a physically active lifestyle [34], they may also need to strengthen their self-efficacy before becoming active. Once they are confident in their physical abilities, positive social control may further enhance self-efficacy and promote activity [35]. 

While the relationship between social control and self-efficacy still needs to be examined empirically, there is evidence for a relationship between exercise self-efficacy and *exercise-specific* social support (which I have argued is the same concept as positive social control for exercise) [14], as well as exercise self-efficacy and *general* social support [36] among older adults. Furthermore, positive and negative aspects of social relations may have different relationships to outcomes depending on whether they are examined cross-sectionally or longitudinally [37]. Therefore, future research should examine physical activity's longitudinal relationship to both positive and negative social control, as well as social control's cross-sectional and longitudinal relationship to exercise self-efficacy. 

Contrary to predictions, general perceived support and strain were not associated with physical activity in the current investigation. These results are inconsistent with a study demonstrating that more perceived support and strain were associated with more frequent physical activity among adults [1]. The discrepancy here may be attributed to study design and statistical power. Cotter and Lachman examined data from over 3,000 participants, whereas I examined data from approximately 300 participants in the current investigation. It is possible that with more statistical power the relationships found in the current investigation would have reached statistical significance. In fact, effect sizes for the relationships in the present study, while not statistically significant, are similar to the effect sizes reported by Cotter and Lachman. 

 Perceived support was moderately related to positive social control in bivariate analyses in the present study, consistent with results reported by Helgeson et al. [13] and McAuley et al. [14]. Additionally, perceived strain was moderately related to both negative social control and positive social control. These results are consistent with assertions that strain may share a stronger relationship to social control than support does, particularly from the perspective of the recipient of social influence [1]. Social partners, then, should be encouraged to be careful to remain positive in their interactions with social control targets. Positive social control is more likely to elicit positive affect, which leads to behavior change without also eliciting negative affect [16]. Thus, positive social control attempts have greater potential for success with fewer negative repercussions. 

When evaluating suggestions based on the present results, it is important to consider the study's strengths and limitations. Regarding the limitations, variables were all examined using a self-report method. Thus, some of the shared variance can be accounted for by the method of data collection. Second, the present study may have lacked sufficient power to corroborate the statistical significance of relationships that have been found using larger samples [1]. Most importantly, the present investigation is based on cross-sectional data. Thus, conclusions regarding causal relationships cannot be made. Furthermore, age differences may be attributed to cohort effects rather than developmental effects. For example, the older adults sampled, particularly the older women, may have approached physical activity differently than the younger adults due to generational differences in attitudes, beliefs, and normative behaviors. While care was taken when collecting the present data to describe physical activity in detail at the outset, older and younger adults may have responded to questions regarding physical activity according to preconceived definitions and thus the results must be interpreted with caution. 

The current study also had a number of strengths. First, perceived support and strain and positive and negative social control were all examined simultaneously, making comparisons between different types of social interaction possible. Indeed, this was the first study to examine perceived strain's relationship to social control. Results from the present investigation suggest that social control may be a more efficacious avenue to explore for physical activity promotion than general support and strain. Second, the use of younger and older adult samples allowed for age comparisons, which revealed different patterns of relationships between variables for younger and older cohorts. Finally, the present sample contained a high percentage of minority participants who are typically underrepresented in research. Thus, the results of the present study are generalizable to a broad population of healthy younger and older adults. 

The results of the current investigation demonstrate how and for whom social control is associated with physical activity. However, longitudinal work must be completed in order to determine the causal direction of these relationships. This is especially important given that previous research shows that social influence variables have different cross-sectional and longitudinal relationships to outcomes [36]. Furthermore, experience time sampling designs, such as daily diary studies, would be useful to determine how daily interactions influence physical activity on that day or in a given week. Daily diary designs would be particularly informative for understanding how, when, and from whom social control might promote physical activity [38]. Finally, self-efficacy plays an important role in behavior adoption [39] and is influenced by the social environment [40]. Therefore, future work should examine the relationship of social control to self-efficacy.

In conclusion, health professionals and intimate social partners should be discouraged from using negative social control strategies because of the potential to induce negative affect, because these strategies may be ineffective for women and younger men, and because these strategies may be counterproductive for older men. Instead, it might be more appropriate to use positive social control strategies, particularly for younger male targets, in order to maintain feelings of support and positive affect while encouraging health behavior change. On the other hand, alternative strategies should be pursued for improving the health and fitness of women and older adults. For example, bolstering self-efficacy may provide an efficacious strategy within these populations.

## Figures and Tables

**Table 1:** Summary of participant characteristics.

	Total Sample		Younger Adults		Older Adults	
	(*N* = 371)		(*N* = 205)		(*N* = 166)	
Variable	*N*	%	*N*	%	*N*	%
Sex						
Male	171	31.7	56	27.9	60	36.1
Female	252	68.3	145	72.1	106	63.9
Race/Ethnicity						
White	183	51.3	81	41.5	101	63.1
African American	41	11.5	17	8.7	24	15.0
Mexican American	43	12.0	31	15.9	11	6.9
Native American	7	2.0	4	2.1	3	1.9
Asian or Pacific Islander	46	12.9	42	21.2	4	2.5
Other	14	3.9	10	5.1	4	2.5
Multiracial	23	6.4	10	5.1	13	8.1
Marital Status						
Married	61	17.0	12	6.0	49	31.4
Separated	6	1.7	0	0	6	3.8
Divorced	42	11.7	0	0	42	26.9
Widowed	45	12.5	1	.5	43	27.6
Never Married	205	57.1	188	93.5	16	10.3
Education						
No school/some grade school	2	.6	0	0	2	1.3
Junior high school	7	2.0	0	0	7	4.6
Some high school	11	3.1	0	0	11	7.2
GED	4	1.1	0	0	4	2.6
High school diploma	26	7.3	0	0	26	17.1
1-2 years of college	130	36.7	109	54.2	20	13.2
3 or more years of college	64	18.1	54	26.9	10	6.6
Associate's degree	49	13.8	32	15.9	17	11.2
Bachelor's degree	27	7.6	6	3.0	21	13.8
Some graduate school	11	3.1	0	0	11	7.2
Master's degree	19	5.4	0	0	19	12.5
Professional degree	4	1.1	0	0	4	2.6
Annual Income						
Less than $10,000	91	27.2	57	29.1	34	24.8
$10,001–$20,000	77	23.0	35	17.9	40	29.2
$20,001–$50,000	81	24.2	42	21.4	39	28.5
$50,001–$75,000	33	9.9	23	11.7	10	7.3
$75,000 or more	53	15.8	39	19.9	14	10.2

*Note:* Age ranged from 18 to 42 (*M *= 21.76, SD = 3.56) for younger adults and from 46 to 97 (*M *= 73.44, SD = 11.02) for older adults.

**Table 2:** Correlations between all variables.

		1	2	3	4	5	6	7	8	9	10	11
1	Age	—	−.09	− **.22**	**.34**	− **.21**	−.07	−.05	− **.18**	−.09	−.10	− **.38**
2	Sex		—	−.00	− *.10*	.01	−.07	**.17**	− *.13*	.07	−.08	−.03
3	Race			—	−.08	−.05	−.06	−.08	**.25**	−.03	.08	.03
4	Marital status				—	.00	**.19**	.07	.09	*.12*	−.01	− **.16**
5	Education					—	*.13*	.05	−.04	*.11*	.00	**.18**
6	Income						—	*.12*	−.05	−.01	− **.17**	.01
7	Support							—	−.08	**.34**	.04	.11
8	Strain								—	**.17**	**.52**	.03
9	PSC									—	**.28**	.12
10	NSC										—	−.01
11	Physical activity											—

*Note: P* ≤ .05, *P* < .01. Age was dichotomized such that 0 = younger than 45 and 1 = older than 45, sex was dichotomized such that 0 = male and 1 = female, race/ethnicity was dichotomized such that 0 = White and 1 = all other races, marital status was dichotomized such that 0 = separated, divorced, widowed, or never married, and 1 = currently married, education was dichotomized such that 0 = up to a high school diploma or GED and 1 = attended some college or higher education, and annual household income was dichotomized such that 0 = $20,000 or less and 1 = $20,001 or more. PSC refers to positive social control and NSC refers to negative social control.

**Table 3:** Summary of two HMR analyses predicting physical activity frequency.

	Step 1			Step 2			Step 3		
	*B*	SE	*β*	*B*	SE	*β*	*B*	SE	*β*
Age	−1.25	.18	−.39**	−1.20	.32	−.38**	−1.24	.32	−.39**
Sex	−.30	.20	−.09	−.29	.27	−.09	−.30	.27	−.09
Support	.26	.20	.08	.34	.20	.10*	.34	.20	.11
Strain	−.07	.18	−.02	−.05	.19	−.02	−.00	.19	-.00
PSC	.13	.10	.08	.37	.21	.23	.74	.28	.46**
NSC	−.80	.80	−.07	−.59	.80	−.05	−.55	.80	−.05
PSC *Age				−.46	.19	−.20*	−1.03	.35	−.45**
PSC *Sex				−.04	.21	−.02	−.52	.32	−.26
Age *Sex				−.07	.39	−.02	−.01	.39	−.00
PSC *Age *Sex							.82	.42	.27*

Change stats	Δ*R* ^2^ = .17,			Δ*R* ^2^ = .02,			Δ*R* ^2^ = .01,		
	*F* (6, 266) = 9.16**			*F*(3,263) = 1.99^+^			*F* (1, 262) = 3.87*		

Age	−1.25	18	−.39**	−1.15	.32	−.36**	−1.05	.32	−.33**
Sex	−.30	.20	−.09	−.25	.27	−.07	−.15	.27	−.05
Support	.26	.20	.08	.30	.20	.09	.33	.20	.10
Strain	−.07	.18	−.02	−.00	.19	−.00	.10	.19	.04
PSC	.13	.10	.08	.15	.10	.09	.15	.10	.09
NSC	−.80	.80	−.07	−.21	1.57	−.02	4.04	1.99	.34*
NSC *Age				−2.01	1.45	−.13	−8.14	2.32	−.52**
NSC *Sex				.72	1.49	.04	−5.33	2.33	−.33*
Age*Sex				−.12	.40	−.04	−.13	.39	−.04
NSC*Age*Sex							9.91	2.97	.41**

Change stats	Δ*R* ^2^ = .17,			Δ*R* ^2^ = .01,			Δ*R* ^2^ = .03,		
	*F* (6, 265) = 9.12**			*F*(3,262) = .93^+^			*F* (1, 261) = 11.16**		

*Note:* ^+^ *P* < .10, *P < .05, **P < .01. PSC refers to positive social control, NSC refers to negative social control, *age refers to the interaction with age, and *sex refers to the interaction with sex.
